# Complete excision of giant clavicular hydatid cyst: a case report

**DOI:** 10.1186/s12879-023-08149-4

**Published:** 2023-03-22

**Authors:** Xin Wang, Jinyong Huang, Liping Su, Qian Ma, Chao Ma, Zengru Xie

**Affiliations:** 1 Department of Orthopedics and Trauma, the First Affiliated Hospital of Xinjiang Medical University, Urumqi, Xinjiang China; 2 Department of Pathologyathology, the First Affiliated Hospital of Xinjiang Medical University, Urumqi, Xinjiang China; 3 Department of Orthopaedics, the Fourth Clinical Medical College of Xinjiang Medical University, Urumqi, Xinjiang China; 4 Key Laboratory of High Incidence Disease Research in Xingjiang (Xinjiang Medical University), Ministry of Education, Urumqi, Xinjiang China; 5 Xinjiang Clinical Research Center for Orthopedics, Xinjiang Medical University, Urumqi, Xinjiang China

## Abstract

**Background:**

Echinococcosis, also known as hydatid disease, is a zoonotic parasitic disease prevalent in pastoral areas, mainly involving the liver and lungs, and less frequently the bones and surrounding soft tissues. Diagnosis and treatment of bone hydatid disease is a challenge, and because of the insidious course of the disease, the lesions are often widely disseminated by the time patients seek medical attention.

**Case presentation:**

A 29-year-old woman presented with a painless mass that was gradually increasing in size in the cervical thorax. Imaging revealed an enlarged clavicle with multiple bone cortical defects and the existence of cysts in the soft tissues surrounding the clavicle, for which complete excision of the clavicle and the attached cysts was performed. There was no recurrence of the cyst within one year after the operation, and the patient felt well and had normal shoulder joint movement.

**Conclusions:**

Bone hydatid may appear in bones throughout the body, and cysts that leak from the bone into the surrounding soft tissues may spread at a relatively rapid rate. Prompt surgical removal of the affected bone and surrounding cysts is necessary for treatment.

## Introduction

Echinococcosis is a zoonosis caused by parasitic infections of the genus Echinococcus, which may affect tissues and organs in various parts of the body. The infection rate of bone tissue is low, accounting for only 0.5% ~ 4% of the total number of cases, but it is very destructive [1]. Spine, femur and humerus were the most frequently reported affected sites. However, in addition to these common bone parts, bone hydatid invasion of other parts of bone tissue is less mentioned. Here we report a rare case of clavicular involvement. This report is written with reference to CARE guidelines [2].

## Case report

**Fig. 1:**
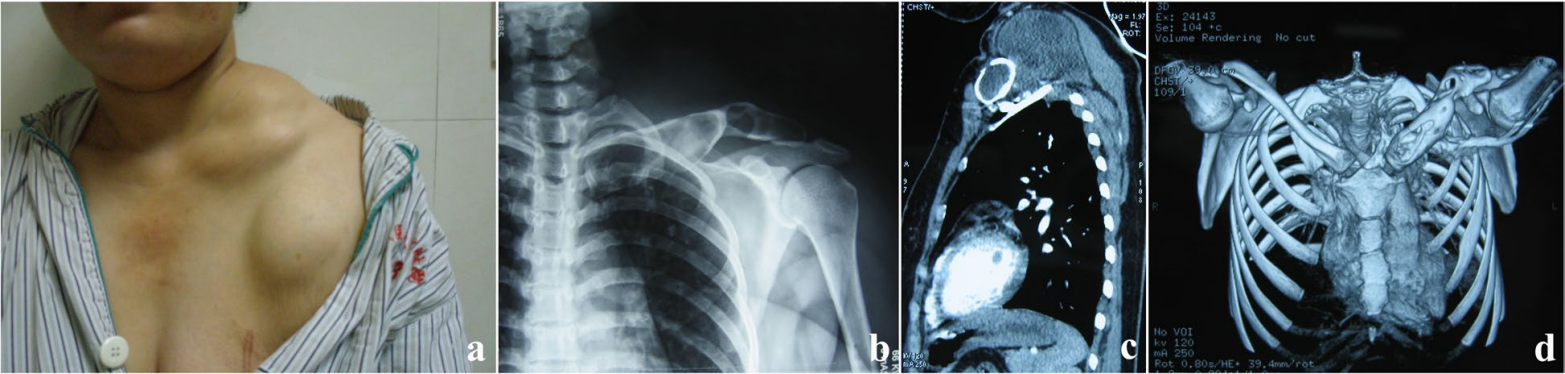
Preoperative appearance and imaging findings of lesions

**Fig. 2:**
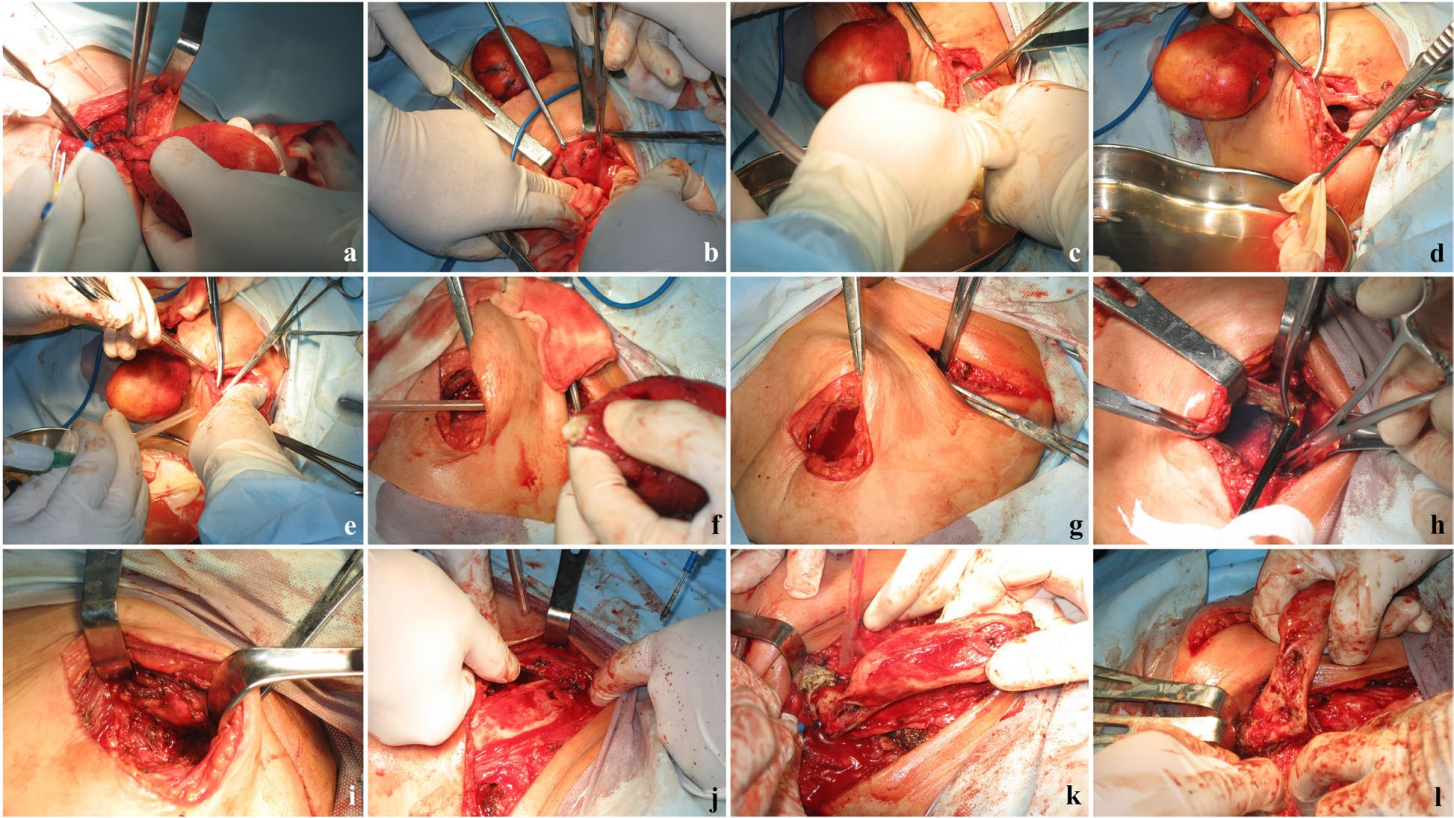
Surgical procsedure and intraoperative performance

**Fig. 3:**
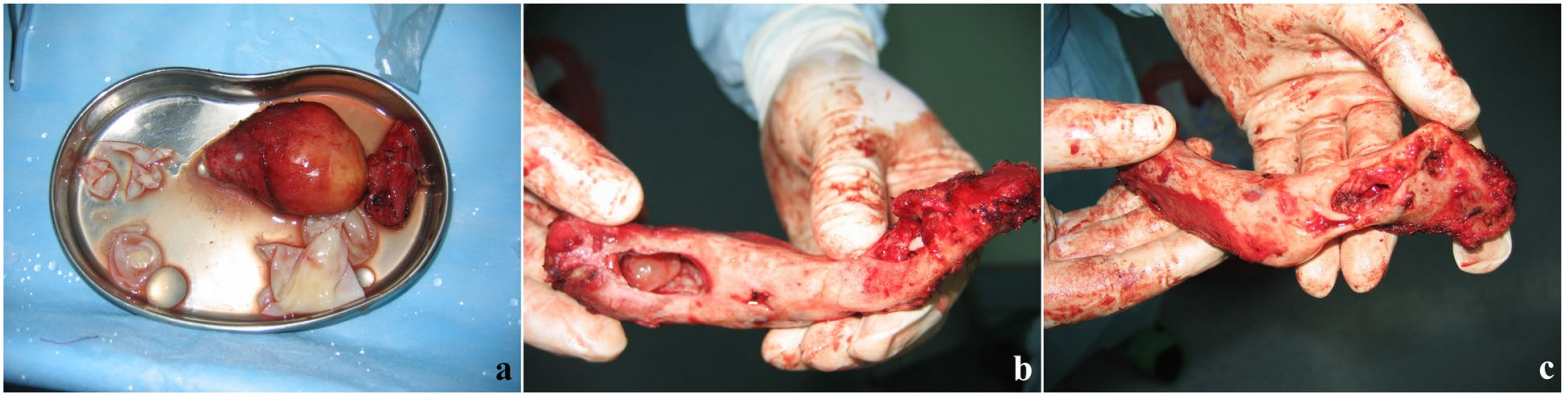
Excised specimen appearance

**Fig. 4:**
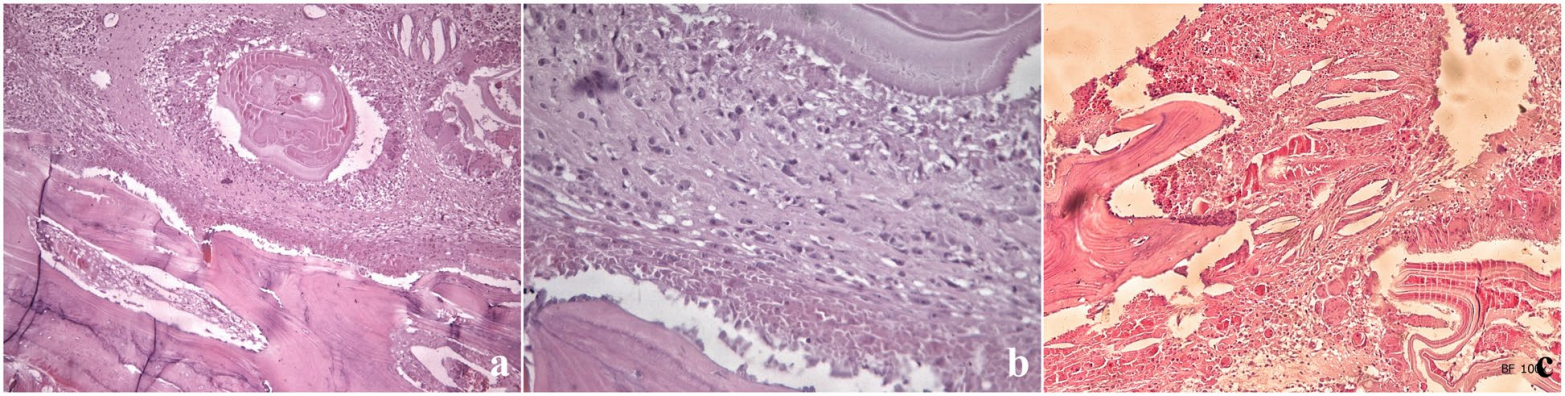
Pathological manifestations of skeletal specimens (4a:HE 100 × , 4b:HE 400 × , 4c:HE 100 ×)

The patient was a 29-year-old female, a herder by occupation. The place of residence was a traditional husbandry area. About 3 months before admission, the patient developed a mass on the left neck with no obvious incentive, with a diameter of 2 cm and no obvious tenderness. The mass gradually increased within 3 months, and by the time of admission, the mass had grown to a size of 6 × 5 cm. One month after the appearance of the mass in the neck, the patient found that a mass with similar characteristics also appeared in front of the left shoulder joint, and gradually increased to an area of 5 × 4 cm on admission (Fig. 1a). The patient had no other systemic specific complaints or positive signs, no family history, and was admitted to the hospital 5 years ago for surgery for a breast abscess. Diet and sleep were normal and there were no manifestations of wasting disease. X-ray and CT examination found that the patient's cervical vertebrae were mildly lateralized to the affected side, and the left clavicle showed swelling bone destruction, combined with cystic masses in the surrounding soft tissues, considering the possibility of echinococcus granulosus cyst (Fig. 1b, d). There were sporadic nodules in both lungs of the patient, some of which were cystic, and the rest were calcified (Fig. 1c). Consider that the patient is more likely to suffer from pulmonary echinococcus. Since there was no significant discomfort, the patient decided to treat the skeletal lesion first and treat the pulmonary hydatid with medication and follow-up. Serological examination (colloidal gold) suggests that the patient's serum is positive for anti-Echinococcus granulosus natural antigen antibodies: anti-EgCF antibodies( +), anti-EgP antibodies( +), anti-EgB antibodies( +), anti-Em2 antibodies(-). There were no specific manifestations in the rest of the patient's blood examinations. After discussion by the medical team, it was decided to treat the patient with surgery and remove the clavicle completely. Surgery process: After general anesthesia, the patient was placed in a supine position with a pillow to elevate the shoulder of the affected side. A horizontal incision of about 10 cm in length was made with the cyst at the front of the shoulder joint and below the outer clavicle (hereinafter referred to as the lateral cyst) as the center, and the skin and dermis were incised layer by layer. A cyst with a size of about 8 × 6 × 5 cm was seen under the skin. blunt dissection (Fig. 2a). Subsequently, the same surgical incision was made with the cyst in the anterior chest wall and the medial clavicle as the center. Exploration revealed a cyst about 10 × 4 × 3 cm in size (hereinafter referred to as medial cyst), wrapping the medial side of the clavicle and extending to the sternoclavicular joint (Fig. 2b). Cut the surface of medial cyst and see several daughter cysts of different sizes inside. Remove the daughter cysts one by one and then remove the remaining external cyst (Fig. 2c, d, e). After that, the lateral cyst was completely removed from the root (Fig. 2f). Careful exploration showed that there were no residual cysts in the subcutaneous cavities on both sides, and the lateral and medial cysts were not connected under the skin. The incision was soaked in 20% hypertonic saline for 30 min (Fig. 2g). Next, separate the adhesion around the clavicle, expose and disconnect the acromioclavicular joint and sternoclavicular joint, and pay attention to protecting the branches of the supraclavicular nerve (Fig. 2h, i, j, k). Pull out the complete clavicle from the medial incision (Fig. 2l), rinse with hypertonic saline again, and then close the surgical wounds on both sides layer by layer. Inspection of the resected cyst and clavicle. An increase in clavicle volume is seen, with several bony cortical defects in the anterior aspect of the sternal end, the anterior aspect of the acromion end, and the area where the posterior subclavian groove joins the conoid tubercle, with significant local thinning of the bone cortex of the lesion and several daughter cysts visible inside (Fig. 3a,b,c). The excised bone tissue specimens are sent for pathological examination. Foreign body granuloma formation is observed next to the bone cortex, and the characteristic endocyst structure of the hydatid cyst is seen within the local granuloma, including the outer cell-free laminar compression-like structure and the inner germinal layer, with fibrous tissue proliferation and histiocyte aggregation in the background (Fig. 4a,b,c). The Patient's postoperative subcutaneous drainage device was removed as scheduled, and the wound healed well without secondary infection. The patient was given oral albendazole at 20 mg/kg per day according to body weight in 2 oral doses for 1 month after surgery, for a total of 5 courses of treatment with a 1-week interval. The patient was instructed to perform functional exercise of the left shoulder 2 weeks after surgery. At the one-year postoperative follow-up, no new cysts were found locally. The patient expressed satisfaction with the treatment effect. Except for the inability to engage in prolonged or strenuous weight-bearing activities, the patient believed that the function of her upper limb could meet the basic needs of life.

## Discussion

Echinococcosis is widely distributed in all continents except Antarctica. Western China has a wide distribution of traditional grazing areas and is a high prevalence area for the disease [3]. According to earlier epidemiological results, 5–30% of the population in western China may be directly threatened by hydatid disease [4]. Risk factors for the disease include engagement in animal husbandry (livestock raising of sheep, cattle, horses and camels) and contact with domestic or wild canids. In some areas, risk factors also included exposure to contaminated water and food [5]. The living environment and occupation of the patient in this case were consistent with a typical population susceptible to hydatid disease.According to relevant studies, the most common site of infection for bone hydatid is the spine. The proportion of other skeletal involvement in the total bone hydatid disease varies from study to study. For example, one study mentioned that, in addition to the spine, the femur, tibia, humerus, skull, and ribs were the most frequently involved sites [6]. Other studies mention the femur, pelvis, humerus, ribs, and tibia as the second most susceptible sites after the spine [7]. A typical hydatid cyst structure consists of a fibrous adventitia, and an endocyst consisting of a laminar and germinal layer. The most direct evidence of progressive echinococcosis infection is if protoscolex derived from fertile cysts can be found in the suspected cystic lesion [8]. The initial lesion of an bone hydatid cyst is usually located in the epiphysis and subsequently spreads in the direction of least resistance within the bone tissue. Due to the hardness of the bone, the adventitia is often not formed, but grows infiltrated as a plural daughter cyst [9]. Clavicle, femur and humerus belong to long bone, which is composed of cancellous bone, wrapped by bone cortex, and has bone marrow cavity. The volume of the bone marrow cavity of the clavicle is relatively large [10], and the abundant blood supply inside provides nutrition and growth space for echinococci, which may be the reason for the occurrence of this case. Conventional laboratory tests in patients with bone hydatid are usually normal and serological examination is the more specific diagnostic method, but due to the erratic detection rate of the experiment [11, 12], pathological examination of intraoperative specimens is still widely used in clinical work for the final diagnosis. The typical pathological presentation is a fibrotic adventitia encapsulated by granulomatous tissue or an endocyst in a hyaline folded state, and in less frequent cases the protoscolex can be directly observed [13]. If the characteristic plural white cystic structures are found intraoperatively, it is highly suspicious of echinococcosis. Although the abnormalities of the bones are very obvious on imaging, the patient does not feel significant discomfort until the local mass appears. Bone hydatid often undergo a long incubation period after infection by the bloodstream route until the onset of symptoms [14]. When a large cyst appears with a peri-spinal area, the patient could be unconsciously and chronically in an unnatural forced postural change due to abnormal sensation or pulling of the focal tissue, as the imaging suggests to us. Unlike the spinal and pelvic areas where the hydatid are susceptible, cysts located in the long bones rarely cause significant neurological compression [15]. In addition to mild swelling and pain as early symptoms, many cysts located in the long bones of the extremities are found incidentally with an externally induced pathological fracture as the first diagnostic factor or, as in this case, due to erosion of the bone cortex and extension of the unrestricted cyst into the subcutaneous and surrounding tissues [16, 17]. Therefore, it is difficult to diagnose and treat long bone hydatid disease in advance [18]. By the time it is detected, the cyst may have spread widely through the bone marrow cavity and local lesion excision or internal fixation placement often ends in failure [19, 20]. In order to completely remove the lesion, complete resection of the entire bone, bounded by the joint, is often required [21, 22]. The clavicle plays a supportive and protective role in the body, but the need for it in upper extremity movement remains controversial [23, 24]. Lesions involving the clavicle, like malignancies, are sometimes opted for complete removal of the clavicle, and the need for postoperative clavicle reconstruction remains unclear [25]. In the present case, the patient's ability to perform nonmanual work was not affected, perhaps demonstrating that resection of the clavicle for complete removal of the lesion is worth advocating. The intraoperative specimen shows a uniform enlargement of the clavicle, with rupture of the proximal and distal bone cortices after erosion and outflow of the protoscolex from the medullary cavity, allowing the cyst to spread to the surrounding subcutaneous tissue. In some cases, a period of perioperative chemotherapy prior to surgery has been chosen to reduce the activity of the cysts and to reduce the probability of intraoperative dissemination and recurrence [26, 27]. However, our cases demonstrate that the rate of local infection spread caused by leakage of broken bone is alarming due to the lax subcutaneous tissue of the neck and anterior chest wall. The clavicle is adjacent to the neck, thorax and mediastinum, and the nearby anatomy is very complex, containing organs, blood vessels and nerves that maintain vital physiological functions, and we believe that immediate treatment of the infected clavicle and cysts within the surrounding tissues is necessary. When performing a hydatid cyst removal procedure, keeping the cyst intact is essential to prevent the spread of the protoscolex. However, when a large hydatid cyst encapsulates bone, we have to incise the adventitia, remove all the endocyst and then remove the affected bone tissue, almost inevitably causing a portion of the endocyst to rupture in the process. Our experience again demonstrates that intraoperative hypertonic saline immersion can effectively inactivate the protoscolex within the occulted cyst and surrounding tissues, avoiding local recurrence of infection. In conclusion, our experience demonstrates that bone hydatid may present in rarer, more dangerous sites and must be managed with prompt and effective surgical management.

## Data Availability

The datasets analyzed during the current study are available from the corresponding author on reasonable request.
